# Gestational trophoblastic neoplasia: experience at Salah Azaiez Institute

**DOI:** 10.11604/pamj.2019.33.121.13897

**Published:** 2019-06-17

**Authors:** Rim Batti, Amina Mokrani, Haifa Rachdi, Henda Raies, Omar Touhami, Mouna Ayadi, Khadija Meddeb, Feryel Letaief, Yosra Yahiaoui, Nesrine Chraiet, Amel Mezlini

**Affiliations:** 1 Department of Medical Oncology at Salah Azaiz Institute, Tunis, Tunisia; 2 “C” Department of Obstetrics and Gynecology, Tunis Maternity and Neonatology Center, Tunis, Tunisia

**Keywords:** Gestational trophoblastic disease, choriocarcinoma, outcomes, prognosis

## Abstract

Gestational trophoblastic disease (GTD) develops from abnormal cellular proliferation of trophoblasts following fertilization. It includes benign trophoblastic disease (hydatidiform moles (HM)) and the malignant trophoblastic diseases or gestational trophoblastic neoplasia (GTN). The frequency of the GTD in Tunisia is one per 918 deliveries. The aim of this study is to analyze the clinical characteristics, treatment and outcomes of GTD at Salah Azaiez Institute (ISA). Medical records of women diagnosed with GTD at ISA from January 1^st^, 1981 to December 31^st^, 2012 were retrospectively reviewed. FIGO score was determined retrospectively for patients treated before 2002. One hundred and nine patients with GTN were included. Patients presented with metastases at 43% of cases. The most common metastatic sites were lung (30%) and vagina (13%). Fifty six (56 (51%) patients had low-risk and 21 (19%) cases had high-risk, the FIGO score was not assessed in 32 cases. After a median follow-up of 46 months, 21 patients were lost to follow-up, 12 patients died, 19 progressed and 8 relapsed. At 10 years, the OS rate was 85% and the PFS rate 79%. OS was significantly influenced by the presence of metastases at presentation (M0 100 % vs. Metastatic 62 %; p < 0.0001), FIGO stage (I-II 100% VS 61% and 65% for stage III and IV; p < 0.001), FIGO score (low-risk 99 % vs. high-risk 78 %; p < 0.001). GTN is a significant source of maternal morbidity with increased risk of mortality from complications if not detected early and treated promptly.

## Introduction

Gestational trophoblastic disease (GTD) develops from abnormal cellular proliferation of trophoblasts following fertilization. It includes benign trophoblastic disease (hydatidiform moles (HM)) and the malignant trophoblastic diseases or gestational trophoblastic neoplasia (GTN). The frequency of the GTD in Tunisia is one per 918 deliveries [1]. The aim of this study was to analyze the clinical characteristics, treatment and outcomes of GTD at Salah Azaiez Institute (ISA).

## Methods

Medical records of women diagnosed with GTD at ISA from January 1^st^, 1981 to December 31^st^, 2012 were retrospectively reviewed. Patients with incomplete records were excluded from the study. Disease diagnosis, treatment, follow-up data, overall survival (OS) and progression free survival (PFS) were analyzed. FIGO score was determined retrospectively for patients treated before 2002. The data processing and analysis were carried out using the SPSS software version 20. Survival probabilities were estimated using Kaplan-Meier method. 126 patients were reported and included during 31 years (1981-2012). Of these cases, 17 (13%) were diagnosed hydatidiform mole and were excluded from this study. One hundred and nine (109) patients presented with GTN and were included.

## Results

During the study period, 109 patients presented with GTN and were included. Their ages ranged between 18 and 53 years with an average of 34 years. Consanguinity was found in 33 cases (45% first degree). The median delay to diagnosis was 3 months (0-36). Sixty eight patients presented with amenorrhea followed by bleeding per vaginally or abortion followed by irregular bleeding per vaginally. GTN occurred after full-term pregnancy in two cases (after one and 14 months). Initial presenting features and reproductive history are summarized in Table 1. Performance status (PS) was = 0 in 35% (38 pts), PS = 1 in 31% (34 pts), PS = 2 in 11% (12 pts) and PS = 3 in 1 case. Ultra sound imaging was performed in all cases, only 72 reports were available. The typical snow storm appearance was described in 29 cases, heterogeneous mass in 30 cases (mean tumor size = 68 mm), increase in uterine size in 10 cases and there was an intraperitoneal effusion in three cases. Histological evidence was obtained in 66 cases. It was obtained by suction dilation and curettage in 41 cases, hysterectomy specimen in 16 cases and a biopsy of a metastasis in nine cases. Histology confirmed 43 cases of choriocarcinoma and 23 of invasive mole. In the other cases the diagnosis was retained on clinical, biological and radiological data. At diagnosis, 53 patients (48%) had localized disease (M0), whereas 43 had metastases and 13 were classified Mx. The most common metastatic sites at initial diagnosis were the lungs (33 cases), and vagina (15 cases). Three patients had brain metastasis, one patient hepatic metastasis, one patient had a sub diaphragmatic mass of 12 cm, one patient had suspicious pelvic nodes and one patient had a biceps muscle metastasis. The mean βhCG level before treatment was 152 170 UI/l (median 10 068). According to FIGO stage, 56 patients were stage I, 4 patients were stage II, 30 patients were stage III and 8 patients stage IV. The FIGO stage was not evaluable in 11 cases. According to FIGO 2002 scoring system, 56 cases were scored low risk and 21 high risk, the FIGO score was not evaluable for 32 patients.

**Table 1:** Initial presenting features and reproductive history

	N
**Age at first intercourse**	
Median	22
Min	15
Max	32
**Age at menarche**	
Mean	13
Min	10
Max	17
**Past medical history**	
Prior Hydatidiform mole	11
Prior spontaneous miscarriage	50
Voluntary interruption of pregnancy	7
**Gravidity**	
0	4
1	6
2	9
≥3	60
Median	4
**Parity**	
0	15
1	12
2	9
≥3	51
Median	3
**Number of children**	
0	11
1	12
2	11
≥3	45
**Contraception**	
Estrogen/progestin	14
intrauterine device	5
No contraception	9
Unkown	81
**Initial presenting features**	
Abnormal vagina bleeding	68
Abdominal pain	6
Metastases	8
During the monitoring after molar pregnancy	10

**Chemotherapy:** in the low-risk group, 24 patients received methotrexate (MTX) and 6 patients received ACT-D. Among high-risk patients, 13 received Cisplatin based regimen associated with actinomycin or adriamycin and etoposide, 1 patient did not receive cisplatin because of renal failure, 1 patient received BEP regimen and one patient received EMACO regimen. Patients treated before the WHO scoring system received different chemotherapy regimen, the decision of the type of chemotherapy was based on only clinical features. When retrospectively assessed, we noted that in four cases of high risk FIGO score patients received single agent chemotherapy and 24 patients with low-risk received combination agent chemotherapy. Treatment modalities are detailed in Table 2. Treatment toxicity totally happened in 50 patients. Grade 2 stomatitis was observed in 19 patients treated with MTX. There were two events of transient generalized skin rush after MTX single agent treatment. Five (4%) patients had grade IV toxicity and four patients died due to chemotherapy toxicity, all of them happened after cisplatine based chemotherapy. One patient had renal failure, two patients had grade IV febrile neutropenia, one patient had hepatic toxicity and one patient died after anaphylactic reaction after cisplatine perfusion.

**Table 2:** Summary of chemotherapeutic protocols. Legend: CRI: complete response to initial chemotherapy

Disease category/ CT	Number of patient	CRIN
**Low risk**		
Single agent Methotrexate	24	17
Single agent Actinomycin	6	4
**Combination chemotherapy**		
MTX-vincristine	6	6
Actinomycin-etoposide	16	8
Cisplatin-adriamycin-etoposide	1	1
Cisplatin-actino-etoposide	1	1
Unkown	2	
**High Risk**		
**Combination chemotherapy**		
Cisplatin-actinomycin-etoposide	8	6
Cisplatin-adriamycin-etoposide	3	2
Cisplatin adriamycin	1	unkown
Etoposide-actinomycin	2	0
Cisplatin actinomycin	1	0
BEP	1	1
EMACO	1	
**Single agent CT**		
Actionomycin	2	0
Methotrexate	1	0
Unkown	1	
**Patients with not assessed FIGO score**		
Single agent CT Methotrexate Combination CT	8	
Actinomycin-etoposide	5	
Methotrexate-oncovin	8	
Actinomycin-vinblastin	2	
Methtrexate-vinblastine	1	
EP/EMA	1	
BEP	1	
Cisplatin-actino-etoposide	1	
Cisplatin-actinomycin-bleo-velbe-Endoxan	1	
Unkown	4	

**Surgery:** eighteen patients underwent initial surgery, 4 patients had uterine perforation and 14 had excessive uterine bleeding. One patient had initial surgery for a biceps muscle metastasis. Five patients were operated after chemotherapy for residual disease.

**Radiotherapy:** two patients had radiotherapy, one for brain metastasis and the other a hemostatic radiotherapy for a bleeding vaginal metastasis.

**Figure 1:**
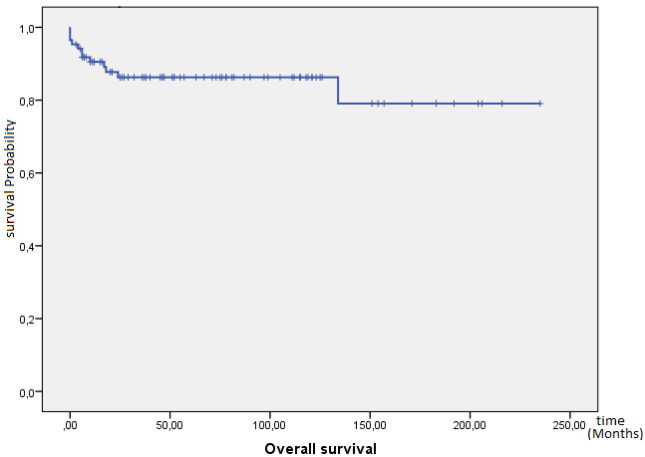
Kaplan-Meier estimates of overall survival

**Figure 2:**
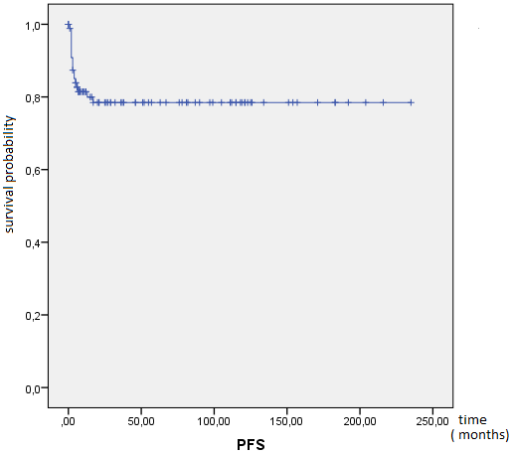
Kaplan-Meier estimates of progression free survival

**Figure 3:**
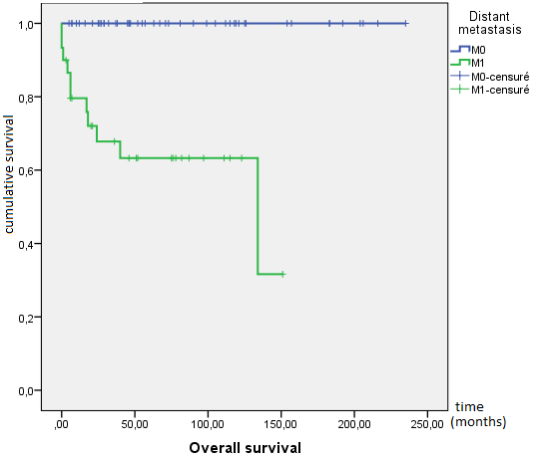
Kaplan-Meier estimates of overall survival by distant metastasis

**Figure 4:**
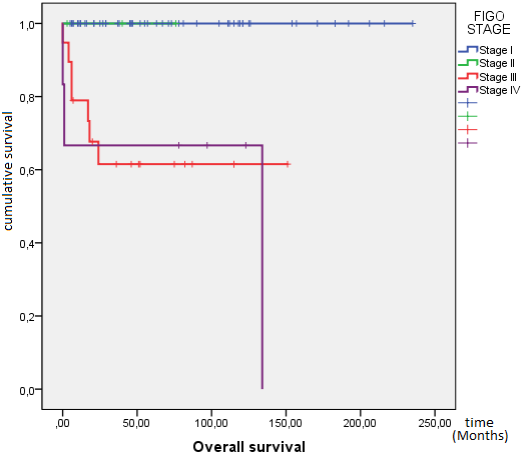
Kaplan-Meier estimates of overall survival by FIGO stage

**Figure 5:**
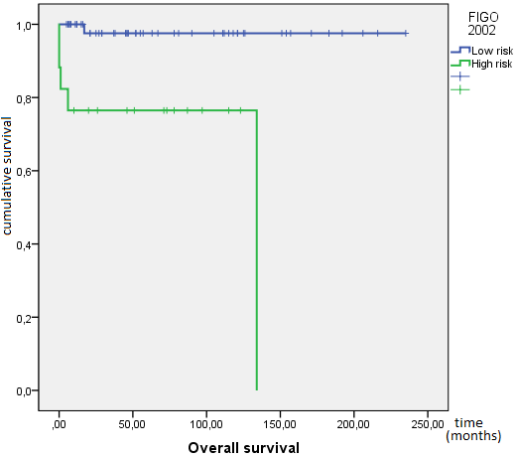
Kaplan-Meier estimates of overall survival by FIGO prognostic score

**Outcome and follow up:** the response to treatment was evaluated during follow-up by clinical examination, ßhCG levels and imaging as and when required. Twenty one (21) patients were lost to follow-up and were excluded from survival analysis. After a median follow-up from initial diagnosis of 46 months, 72 patients achieved complete response, 12 patients died (eight due to disease progression and four due to chemotherapy toxicity), 19 patients progressed to initial chemotherapy and eight patients relapsed. At 10 years, OS was 82%. The mean OS was 196 months IC95% (175-218) (Figure 1). Mean PFS was 185 months IC95% (164-205), at 10 years PFS rate was 79% (Figure 2). OS was significantly influenced by the presence of metastases at presentation (M0 100% vs. Metastatic 62%; p < 0.0001) (Figure 3). Patients with advanced stage disease had poor survival when compared to the early stage group (I 100%, II 95% vs. III 62%, IV 62%; p = 0.001) (Figure 4). Patients with high-risk prognostic score compared to the low risk group showed poorer outcome (low-risk 99% vs. high-risk 76%; p = 0.003) (Figure 5). After achieving treatment, 13 conceptions were reported resulting in no molar pregnancy: 3 miscarriage and 10 full-term pregnancies. The mean time to pregnancy after achieving treatment was 37 months (range 8-123 months). Nine patients had full-term healthy births and a still birth in one case. One patient had repeated artificial insemination failure.

## Discussion

GTD is a heterogeneous rare group of clinical conditions characterized by disordered differentiation and/or the proliferation of trophoblastic epithelium. GTN can arise after any type of pregnancy and can develop months or years after prior pregnancy. It affects women in the age group of 20 to 30 years. In our study, the median age was 34 years which is in comparison to peer reviewed published literature [2]. An increased risk of GTN have been linked to hormonal factors such as light menstrual flow, a menarche after 12 years-old, the use of oral contraceptive [3]. Other potential risk factors have also been reported for example; blood group A, past history of hydatidiform mole and maternal age [4]. GTN is a highly vascular disease and taking a biopsy of lesions are extremely risky. Histological confirmation is not essential before commencing chemotherapy. However, it may be useful to confirm diagnosis and obtain genetic analysis. Genotyping will be helpful to determine the causative pregnancy in patients with multiple pregnancies and to distinguish GTN from non gestational tumor [5]. The ßhCG measurement is essential for diagnosis and management of GTD. Some assays can lead to false negative or false positive results because hCG can exist in different forms in patients with GTN. It's essential that the hCG assay can measure all forms of hCG [3]. Since 2002, the management of GTN is guided by the FIGO prognostic scoring. Patients with high risk disease had 0% chance to be cured with a single-agent chemotherapy. One recent study re-evaluated all prognostic risk factors involved in the FIGO scoring system in 813 patients with GTN and proposed a simplified alternative using only five factors [6]. GTN most frequently spreads to the lung. In our study, vaginal metastases were present in 13%. In reported series vaginal metastases are present in 4-30% in GTN [7, 8]. Patients with low-risk GTN can usually be treated successfully with single-agent chemotherapy. In our series of 56 low-risk patients, we observed a complete remission rate of 70% to single-agent Methotrexate and 66% to single-agent Dactinomycin. A randomized clinical trial compared biweekly Dactinomycin versus weekly Methotrexate demonstrated a superior response rate for Dactinomycin over Methotrexate (69 vs. 53% ; p = 0.015) [9].

Retrospective reviews of patients with low-risk GTN treated with single-agent chemotherapy showed significantly higher primary remission rate with Dactinomycin than with Methotrexate regimen [10, 11]. Due to decreased cost and the good tolerance of Methotrexate, especially for not inducing hair loss, Methotrexate was preferred as first line treatment. However a recent study suggest that women with hCG >400,000 UI/l are unlikely to be cured by single-agent chemotherapy and should be treated by multi-agent chemotherapy as first line treatment [12]. Among the patients with high-risk GTN, our data supports the effectiveness of the combination chemotherapy. The preferred regimen in our study was the actinomycin, cisplatin and etoposide French regimen. The complete response rate with this regimen was 94.7% and the five-year OS 97%. This regimen is highly active as first-line and as treatment for persistent/recurrent GTN [13]. Several regimens were developed. EMACO is used worldwide and is the optimal primary treatment because of short term toxicity and effectiveness [14]. A Korean study demonstrated a higher remission rate (90.6%) with EMACO when compared with others regimens [15]. Patients with advanced disease may benefit from initial low dose chemotherapy to reduce early deaths [16]. Our study confirms the lower outcomes in patients with advanced-stage disease, metastatic disease and high-risk prognostic score [11, 17]. The overall worldwide survival rate of low-risk GTN group is 100%, and 80-90% for high-risk GTN group [11]. The overall survival rate for patients with GTN treated at our institute was 85%. The reproductive success rates and gestational complications have become a concern for women treated for GTD. EMACO regimen may induce menopause three years earlier but fertility is not affected and 83% of women became pregnant after Methotrexate or EMACO [3]. The number of studies in the literature regarding this theme is small. The studies highlight that there is no change in fertility [18]. Under close monitoring, patients may conceive six months after achieving complete response [19]. One study demonstrated a slight increase in stillbirth after GTN treatment [20]. In our study, conception occurred resulting in no molar pregnancy, miscarriage and full-term pregnancy.

## Conclusion

GTN is a significant source of maternal morbidity with increased risk of mortality from complications if not detected early and treated promptly. Management of gestational disease should ideally be done in a specialized multi-disciplinary environment.

### What is known about this topic

- Gestational trophoblastic neoplasia are malignant disorders of invasive mole, choriocarcinoma, and the rare placental-site trophoblastic tumour. Overall cure rates can exceed 98%. This success can be explained by the development of effective treatments and centralization of care.

### What this study adds

- Report a Tunisian experience with gestational trophoblastic neoplasia;
- The results of this study can help to encourage a specialized multi-disciplinary environment for the management of gestational disease.

## Competing interests

The authors declare no competing interests.
